# Relationship between amniotic fluid metabolic profile with fetal gender, maternal age, and gestational week

**DOI:** 10.1186/s12884-021-04116-6

**Published:** 2021-09-18

**Authors:** Yahong Li, Yun Sun, Xiaojuan Zhang, Xin Wang, Peiying Yang, Xianwei Guan, Yan Wang, Xiaoyan Zhou, Ping Hu, Tao Jiang, Zhengfeng Xu

**Affiliations:** 1 Center for Genetic Medicine, Women’s Hospital of Nanjing Medical University, Nanjing Maternity and Child Health Care Hospital, 123 Tianfei Road, Nanjing, Jiangsu 210004 P. R. China; 2 Department of Obstetrics, The Affiliated Huaian No, 1 People’s Hospital of Nanjing Medical University, Huaian, Jiangsu 223001 P. R. China

**Keywords:** Amniotic fluid, Metabolomics, Gender, Age, Gestational week

## Abstract

**Background:**

Amniotic fluid (AF) provides vital information on fetal development, which is also valuable in identifying fetal abnormalities during pregnancy. However, the relationship between the metabolic profile of AF in the second trimester of a normal pregnancy with several maternal–fetal parameters remains poorly understood, which therefore limits its application in clinical practice. The aim of this study was to explore the association between the metabolic profile of AF with fetal gender, maternal age, and gestational week using an untargeted metabolomics method.

**Methods:**

A total of 114 AF samples were analyzed in this study. Clinical data on fetal gender, maternal age, and gestational week of these samples were collected. Samples were analyzed by gas chromatography/time-of-flight-mass spectrometry (GC-TOF/MS). Principal component analysis(PCA), orthogonal partial least square discrimination analysis(OPLS-DA) or partial least square discrimination analysis (PLS-DA) were conducted to compare metabolic profiles, and differential metabolites were obtained by univariate analysis.

**Results:**

Both PCA and OPLS-DA demonstrated no significant separation trend between the metabolic profiles of male and female fetuses, and there were only 7 differential metabolites. When the association between the maternal age on AF metabolic profile was explored, both PCA and PLS-DA revealed that the maternal age in the range of 21 to 40 years had no significant effect on the metabolic profile of AF, and only four different metabolites were found. There was no significant difference in the metabolic profiles of AF from fetuses of 17–22 weeks, and 23 differential metabolites were found.

**Conclusions:**

In the scope of our study, there was no significant correlation between the AF metabolic profile and the fetal gender, maternal age and gestational week of a small range. Nevertheless, few metabolites appeared differentially expressed.

## Background

Amniotic fluid (AF) is the fluid that surrounds the fetus in the amniotic cavity, which plays a key role in protecting and providing nutrients to the fetus. Numerous studies have shown that AF contains DNA, RNA, and metabolites that have vital biological functions in fetal development, which can be used to identify any fetal developmental abnormalities [1–3].

Metabolomics is a rapidly advancing field in research and clinical applications following genomics, transcriptomics, and proteomics [4]. The concept of metabolomics was firstly proposed and defined by Nicholson et al. [5] as “the quantitative measurement of the dynamic multiparametric metabolic response of living systems to pathophysiological stimuli or genetic modification”. It explores the changes of endogenous small molecule metabolites(< 1000 Da), such as amino acid, carbohydrate, and organic acid upon the interaction with factors including heredity, environment, diet, drugs, disease, etc. [6, 7]. In the case of organisms, DNA, RNA and proteins are the material basis of biological events or processes, but the event may or may not occur, and the presence of metabolites reflects what has already happened in the course of life. Therefore, the metabolome is more reflective of a person's phenotype.

Metabolomics can be divided into targeted and non-targeted metabolomics according to the pertinence of detection methods. Targeted metabolomics accurately determines specific or several metabolites with similar properties, while untargeted metabolomics conducts a systematic and comprehensive analysis of the entire metabolome of an organism without bias, thus obtaining substantial small-molecule metabolite data [8, 9]. Common detection methods include nuclear magnetic resonance(NMR), liquid chromatography mass spectrometry(LC–MS) and gas chromatography-mass spectrometry(GC–MS) [10]. Numerous types of samples can be analyzed by metabolomics, including blood, urine, amniotic fluid, stool, tissue, cells, dried blood spot, etc. [11–14]. In recent years, metabolomics has helped in making remarkable advancements and achievements in many fields, including biomarker screening, disease diagnosis, determining disease pathogenesis, and drug development [15, 16].

Studies have shown that the composition of metabolites in biological samples may be influenced by factors such as gender, age, diet, lifestyle, environmental, etc. [17–20]. Considering this, AF as a biological sample undergoes changes throughout the gestational week as the fetus develops. However, to date, few studies have examined the factors affecting the metabolomics of AF. The study by Orczyk-Pawilowicz et al. [21] demonstrated that the AF during the transition from the 2^nd^ (15.4 ± 0.96 weeks) to the 3^rd^ (37.7 ± 1.68 weeks) trimester was associated with elevated levels of creatinine, succinate, pyruvate, choline, N,N-dimethylglycine, and urocanate, while a reduction in the levels of amino acids, glucose, and carnitine was observed. However, the impacts of gestational week, fetal gender and maternal age on AF metabolic profile warrant further exploration.

In this study, untargeted metabolomics based on GC-TOF/MS was applied to explore the relationship between fetal gender, maternal age, and gestational week with the metabolic profile of AF in the second trimester, in order to identify potential influencing factors that may provide a foundation for further AF-related metabolomics research in the future.

## Methods

### Samples

From January 2012 to December 2018, a total of 1,859 AF samples were collected prospectively, from pregnant women of similar ethnic backgrounds in the Jiangsu Province. These were residual AF following clinical molecular diagnostic tests in our prenatal diagnostic center, which were stored at -80^0^C. Of these, 114 AF samples were selected for this study, with the inclusion criteria as follows: singleton pregnancy; amniocentesis performed due to advanced age or high risk for Down’s Syndrome following a serological screening in the second trimester of pregnancy; analysis of AF showed normal karyotypes; and no abnormality was identified after birth during the postnatal follow-ups. AF samples were excluded if they were from pregnant women with pregnancy-related disorders, such as hypertension and diabetes.

### Chemical materials and instruments for untargeted metabolomics

Pyridine, methoxyamine HCl, anhydrous sodium sulfate, and fatty acid methyl ester standards (C7–C30, FAMEs) were purchased from Sigma-Aldrich (St. Louis, MO, USA), while N-methyl-N(trimethylsilyl)trifluoroacetamide (MSTFA) with 1% (vol/vol) trimethylchlorosilane (TMCS), dichloromethane, hexane, chloroform, methanol (Optima LC–MS), acetonitrile (Optima LC–MS) and acetone were purchased from Thermo-Fisher Scientific (FairLawn, NJ, USA). Ultrapure water was obtained through a Milli-Q reference system (Millipore, Billerica, MA, USA). GC-TOF/MS (Pegasus HT, Leco Corp., St. Joseph, MO, USA) was equipped with an Agilent 7890B gas chromatography, a Gerstel multipurpose sample MPS2 (Gerstel, Muehlheim, Germany), and a Rxi-5 ms capillary column (30 m × 250 μm i.d., 0.25 μm film thickness, Restek Corporation, Bellefonte, PA, USA).

### Sample processing

The frozen AF samples stored at -80 °C were thawed on ice. Then, they were mixed well and centrifuged at 1,000 g for 3 min at 4 °C. Each 100 μL of AF sample was aliquoted into a pre-cooled Eppendorf tube and 10 μL of the internal standard solution was added. For metabolite extraction, each 200 μL of pre-cooled methanol: chloroform (3:1, v/v) was used. After centrifugation (13,000 g, 20 min, 4 °C), the supernatant was transferred into an auto-sampler vial (Agilent Technologies, Foster City, CA, USA). Quality control (QC) samples were prepared by mixing the remaining supernatant from each AF sample. To remove the chloroform solvent, all the samples were centrifuged for 5 min in a vacuum centrifuge concentrator (Labconco, Kansas City, MO, USA), and then transferred to a freeze dryer (Labconco, Kansas City, MO, USA) and completely lyophilized. Dichloromethane was added to ensure complete dryness of the samples, followed by high-purity nitrogen (Parker Balston, Lancaster, NY, USA) filling in the dried powder at room temperature. Untargeted metabolite analysis was performed on the XploreMET platform (Metabo-Profile, Shanghai, China). Briefly, 50 μL of methoxyamine (20 mg/mL in pyridine) was added to each dried sample at 30 °C for 2 h and then mixing with 50 μL of MSTFA (1% TMCS) containing FAMEs at 37.5 °C for 1 h.

### Chromatographic conditions

Helium (99.9999%) was used as the carrier gas at the constant flow rate of 1.0 mL/min. The injection volume was 1 μL, and the injection and transfer interface temperatures were both at 270 °C. The GC temperature programming was set to 2 min of isothermal heating at 80 °C, followed by 12 °C/min oven temperature ramps at 80–300 °C, 4.5 min at 300 °C, 40 °C/min at 300–320 °C, and 1 min of final maintenance at 320 °C.

### Mass spectrometry conditions

In the full scan mode (m/z 50–500), metabolites were measured using electron impact ionization (70 eV), and the ion source was set at 220 °C. The acquisition rate was 25 spectra/s, and the mass range was 50–500 Da.

### Data analysis

Peak picking, automated baseline denoising, deconvolution, and signal alignment were processed by XploreMET 3.0 software [22, 23]. Each data set was transformed into the comparable data vectors, and MetaboAnalyst 5.0, an open software, was used for multivariate statistical analysis, including the PCA, OPLS-DA and PLS-DA. PCA is an unsupervised classification model, which continuously reduces the dimensions of multi-dimensional data into several main components (PCs) to describe the characteristics of the original data to the furthest extent possible [24]. On the other hand, the PLS-DA model and OPLS-DA model are supervised classification models, which can maximize the differences between groups according to the predefined classification and achieve a better separation effect than PCA [25]. However, supervised analytical models may produce the phenomenon of overfitting [26]. There are two parameters, R^2^Y and Q^2^ in the model, of which R^2^Y measures the goodness of fit and Q^2^ measures the predictive ability of the model. The closer these two values are to 1, the more exemplary the model, with Q^2^ > 0.5 indicating good predictability [27]. A negative value of Q^2^ indicatesthe overfitting of the model, suggesting no significant difference between the groups [28]. Univariate statistical analysis was performed using SPSS 22.0 software (IBM, USA), and *P* < 0.05 was considered statistically significant.

## Results

### Association between fetal gender and AF metabolic profile

The demographics including the number of AF samples, maternal age range, and gestational week range based on gender were outlined in Table 1. A total of 64 maternal AF samples of female fetuses and 50 male fetuses were included. For the female and male fetal groups, the medians of the gestational week were both at 20 (17–24) weeks, while the maternal age was at 31.92 ± 5.20 and 31.00 ± 5.65 years, respectively. When comparing the 2 groups, there were no statistically significant differences in the gestational week (*P* = 0.80) and maternal age (*P* = 0.25).

**Table 1:** Demographic characteristics of subjects in studying the effect of fetal gender on AF metabolic profile

Subject characteristics	Female	Male	*P*-value
Number of samples(n)	64	50	
Gestational week, median	20(17 ~ 24)	20(17 ~ 24)	0.800
Maternal age (years),‾x ± SD,	31.92 ± 5.20	31.00 ± 5.65	0.250

**Fig. 1:**
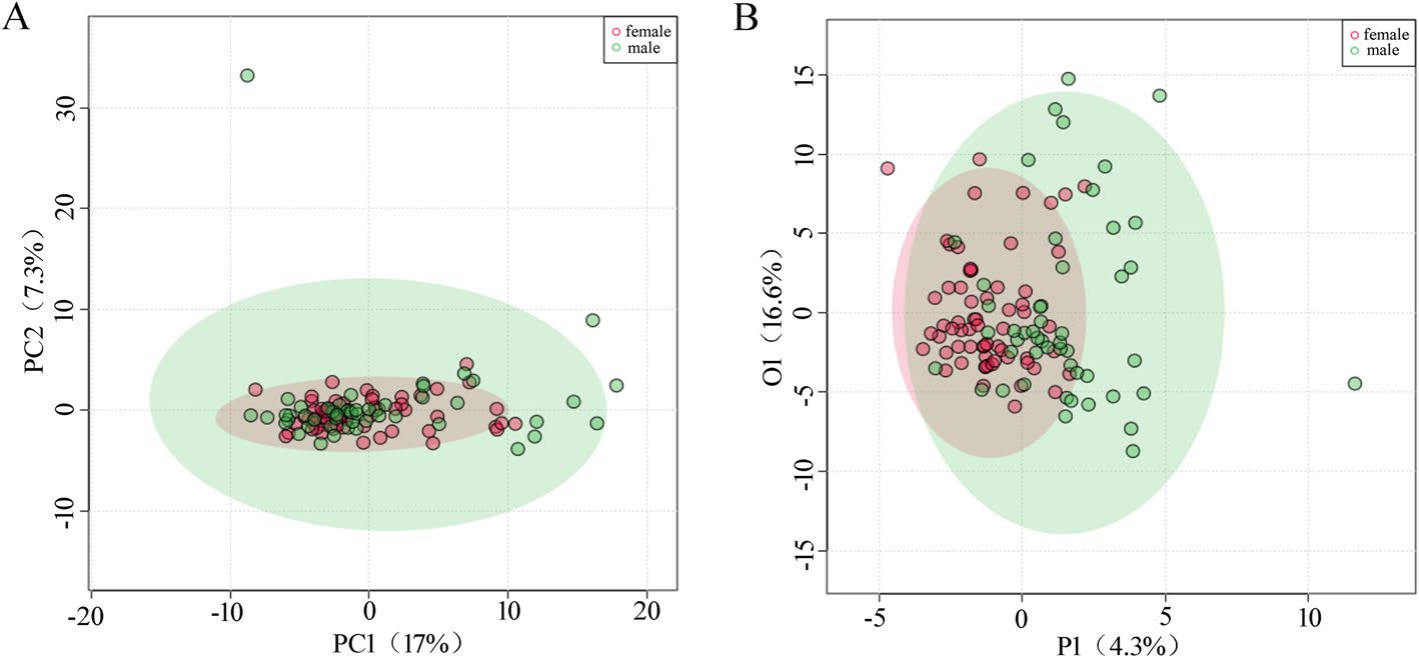
Multivariate analysis of the influence of the fetal gender on the AF metabolic profile. **A** Two dimensional unsupervised PCA analysis; **B** Supervised OPLS-DA, R^2^Y = 0.340; Q^2^ = -0.224. A negative Q^2^ value indicated an over-fitting of the model

In an unsupervised PCA analysis model, there was no significant trend of separation of the metabolic profiles between the female and male fetal groups (Fig. 1A). The OPLS-DA model showed a trend of separation between the two groups, but the parameter Q^2^ value was -0.224, indicating that this trend of separation was attributed to the overfitting of the models rather than the difference in the gender groups (Fig. 1B). These findings suggested no significant association between the difference in the fetal gender and the total metabolic profile of AF.

A total of 265 metabolites were detected in the amniotic fluid, of which 178 were successfully identified. Of these, the proportions of amino acids, organic acids, carbohydrates, nucleotides, lipids, indoles, fatty acids and others were 30%, 25%, 19%, 7%, 3%, 3%, 3%, and 10%, respectively.

Univariate analysis revealed 7 differently expressed metabolites between the two groups, including isoleucine, 3-aminoisobutanoic acid, ribonolactone, fructose 6-phosphate, citric acid, phosphoenolpyruvic acid, and hypoxanthine (Table 2).

**Table 2:** Differential metabolites of male and female fetuses in the maternal AF

Class	Metabolite	HMDB ID	Trend in female	FC	*P*-value
Amino Acids	Isoleucine	HMDB0000172	↑	1.22	0.040
	3-Aminoisobutanoic acid	HMDB0003911	↓	0.83	0.049
Carbohydrates	Ribonolactone	HMDB0001900	↓	0.42	0.029
	Fructose 6-phosphate	HMDB0000124	↑	1.15	0.043
Organic Acids	Citric acid	HMDB0000094	↑	1.17	0.040
	Phosphoenolpyruvic acid	HMDB0000263	↑	1.17	0.017
Nucleotides	Hypoxanthine	HMDB0000157	↓	1.20	0.018

*HMDB* Human metabolome database, *FC* Fold change

### Association between maternal age and AF metabolic profile

The demographics including the number of AF samples, fetal gender composition, and gestational week range based on the maternal age were outlined in Table 3. AF samples were categorized into 4 groups of maternal ages, including 21–25 years, 26–30 years, 31–35 years and 36–40 years, with the numbers of AF samples in each group were 16, 37, 33 and 19, respectively, while the numbers of female/male fetuses were 8/8, 19/18, 20/13 and 11/8, respectively, with no significant difference between the groups. Similarly, no significant difference was demonstrated in the medians of the gestational week between the groups, which were 19.5 (17–24) weeks, 20 (18–24) weeks, 20 (17–23) weeks and 20 (18–23) weeks, respectively.

**Table 3:** Information on fetal gender and gestational week in different maternal age groups

Maternal age(years)	n	Gender (female/male)	*P*-value	Gestational week (week), median	*P*-value
21 ~ 25	16	8/8	0.839	19.5(17 ~ 24)	0.374
26 ~ 30	37	19/18		20(18 ~ 24)	
31 ~ 35	33	20/13		20(17 ~ 23)	
35 ~ 40	19	11/8		20(18 ~ 23)	

**Fig. 2:**
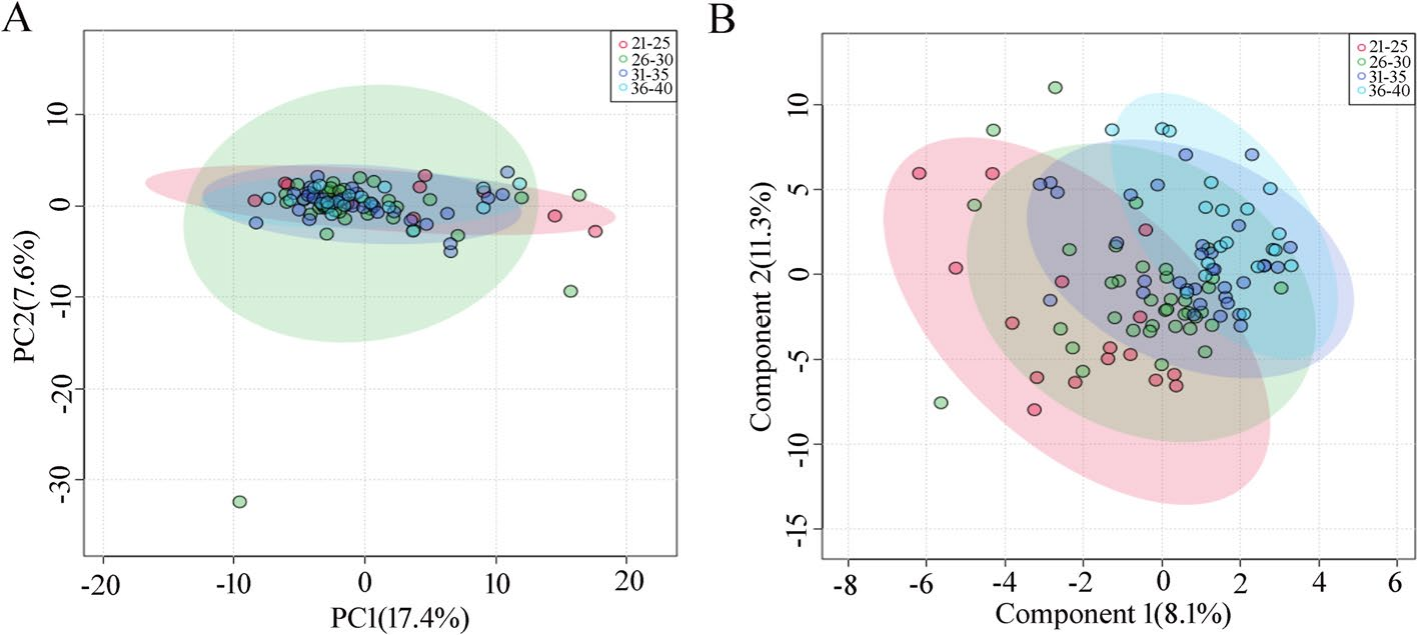
Multivariate analysis of the influence of maternal age on the AF metabolic profile. **A** Two dimensional unsupervised PCA analysis; **B** Supervised PLS-DA, the Q^2^ value of the first three components was-0.22. A negative Q^2^ value indicated an over-fitting of the model

PCA was performed to explore the differences in the metabolic profile between the four groups, which showed no significant trend of separation (Fig. 2A). Similar findings were observed when the PLS-DA model was performed (Fig. 2B). The model parameter Q^2^ value of the first three components was-0.22 and the *P*-value of the permutation test of the model was 0.54. These findings indicated that maternal age had no significant influence on the AF metabolic profile. Among the 178 metabolites successfully identified, only 2-hydroxybutyric acid, guanidinosuccinic acid, erythrose and putrescine were significantly different among these groups (Table 4).

**Table 4:** Differential metabolites of different maternal age in AF

Class	Metabolite	HMDB ID	*P*-value
Organic Acids	2-Hydroxybutyric acid	HMDB0000008	0.007
	Guanidinosuccinic acid	HMDB0003157	0.025
Carbohydrates	Erythrose	HMDB0002649	0.003
Alkylamines	Putrescine	HMDB0001414	0.047

*HMDB* Human metabolome database

### Association between gestational week and AF metabolic profile

A total of 109 samples were selected to explore the association between the gestational week and the metabolic profile of AF, as shown in Table 5. There were 3 gestational week groups, which were 17–18 weeks (12 cases), 19–20 weeks (67 cases), and 21–22 weeks (30 cases), with the numbers of female/male fetuses in each group were 5/7, 39/28, and 18/12, respectively, the difference of which between the groups was not statistically significant. Also, no significant difference was demonstrated between the groups in the medians of the maternal age, which were 28.5 (24–42) years, 31 (21- 42) years, and 31 (24–42) years, respectively.

**Table 5:** Information on fetal gender and maternal age in different gestational week groups

Gestational week (week)	n	Gender (female/male)	*P*-value	Maternal age (year), median	*P*-value
17 ~ 18	12	5/7	0.522	28.5(24 ~ 42)	0.812
19 ~ 20	67	39/28		31(21 ~ 42)	
21 ~ 22	30	18/12		31(24 ~ 42)	

**Fig. 3:**
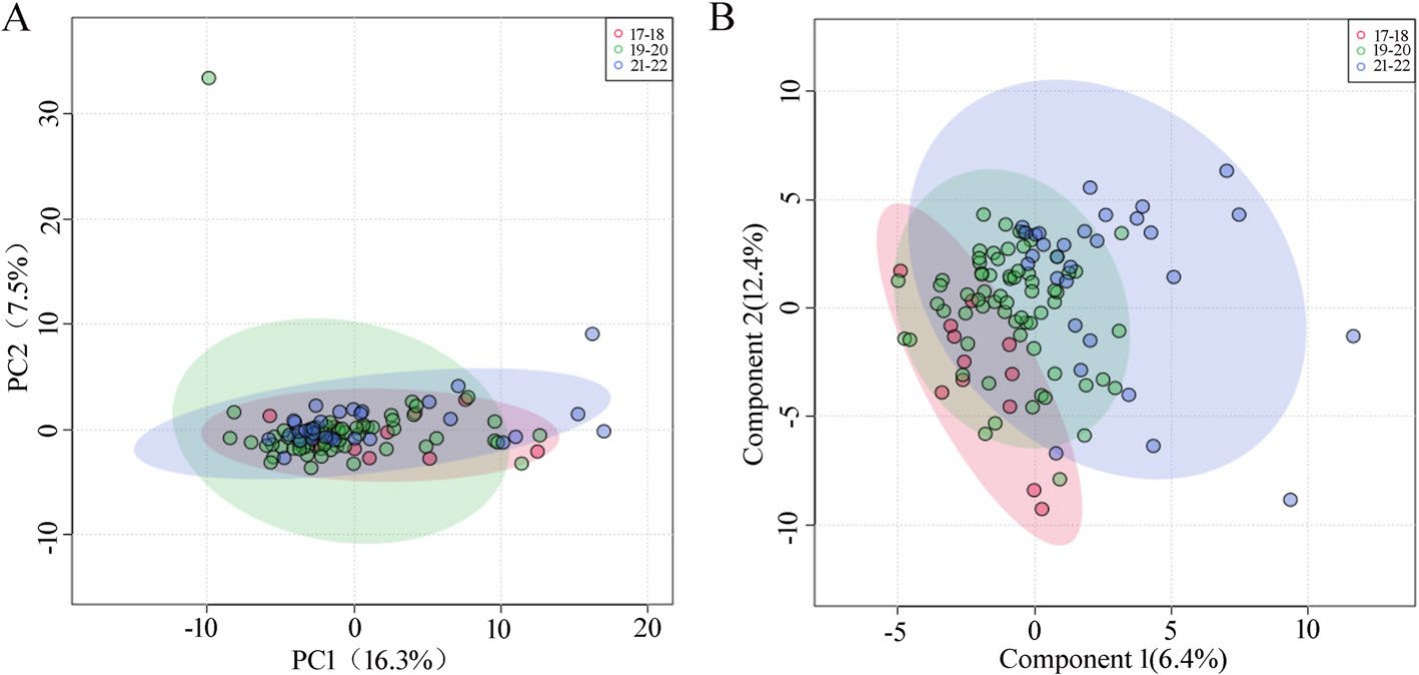
Multivariate analysis of the influence of the gestational week on the AF metabolic profile. **A** Two dimensional unsupervised PCA analysis; **B** In the PLS-DA model, the Q^2^ value of the first three component was -0.14. A negative Q^2^ value indicated an over-fitting of the model

The PCA revealed no significant separation in the AF metabolic profiles among the three gestational week groups (Fig. 3A), which were further validated by the PLS-DA model with a negative Q^2^ value (Fig. 3B). These findings indicated that gestational week had no significant influence on the AF metabolic profile at 17 to 22 weeks, but had an impact on the level of 23 metabolites, as shown in Table 6.

**Table 6:** Differential metabolites in different gestational week

Class	Metabolite	HMDB ID	*P*-value
Amino Acids	Creatinine	HMDB0000562	0.0004
	Creatine	HMDB0000064	0.0013
	Glutamic acid	HMDB0000148	0.0038
	Histidine	HMDB0000177	0.0255
	Threonine	HMDB0000167	0.0244
	Tyrosine	HMDB0000158	0.0128
	N-Acetyl-aspartic acid	HMDB0000812	0.0080
	Glutamine	HMDB0000641	0.0440
Carbohydrates	D-Ribose	HMDB0000283	0.0239
	Ribitol	HMDB0000508	0.0364
	Gluconic acid	HMDB0000625	0.0068
Organic Acids	Homogentisic acid	HMDB0000130	0.0066
	Isocitric acid	HMDB0000193	0.0087
	Ketoleucine	HMDB0000695	0.0252
	Taurine	HMDB0000251	0.0330
Lipids	Hexanoylcarnitine	HMDB0000705	0.0029
	Glycerol 3-phosphate	HMDB0000126	0.0122
	MG(18:2(9Z,12Z)/0:0/0:0)	HMDB0011568	0.0468
Fatty Acids	Pimelic acid	HMDB0000857	0.0003
Indoles	Indoleacetic acid	HMDB0000197	0.0031
Alkylamines	Hydroxylamine	HMDB0003338	0.0061
Alcohols	Myoinositol	HMDB0000211	0.0347
NA	GLUTAMIC	NA	0.0105

*HMDB* Human metabolome database

## Discussion

The innovations of chromatography, mass spectrometry, as well as bio-information technology have enabled metabolomics to develop rapidly. Metabolomics is a growing field of research especially in liver disease, cancer, cardiovascular disease, traditional Chinese medicine, and intestinal microbiota. Loomba et al. [29] have used metabolomics to evaluate the relationship between dose and therapeutic effect of GS-0976 in patients with nonalcoholic steatohepatitis (NASH). Also, Huang et al. [30] have carried out a large prospective serum metabolomic research involving 523 cases of lethal prostate cancer and an equal number of matched controls. In the study, 34 metabolites were associated with lethal prostate cancer, in which dipeptide leucylglycine and three gamma-glutamyl amino acids were associated with an increased risk of lethal prostate cancer. The study by Chen et al. [31] has applied the untargeted metabolomics method to examine and compare serum samples of untreated black hypertensives treated with slow sodium tablets or placebo tablets, and identified β-hydroxyisovalerate and methionine sulfone were significantly increased in the treatment group, indicating that low sodium diet reduces blood pressure. In traditional Chinese medicine, studies have applied metabolomics to explore the components and pharmacokinetics of ginseng [32]. In recent years, research on intestinal microorganisms has been popularized. The study by Hagan et al. [33] has demonstrated the important role of intestinal microorganisms in regulating the body's immunity. All in all, metabolomics is a valuable tool for exploring drug efficacy, screening of tumor markers, studying of pathological mechanisms, drug metabolism and many other aspects.

Metabolomics also has important applications in the studies of abnormalities in pregnant women or fetuses during pregnancy. Bahado-Singh et al. [34] have used NMR to explore the serum profile of pregnant women at 11–13 weeks to predict pre-eclampsia, and the results demonstrated that a combination of citrate, hydroxyisovalerate, glycerol, and methionine produced a better predictive effect (75%). Also, the study by Ciborowski et al. [35] has revealed that serum metabolites in early pregnancy could predict the risk of macrosomia. AF is crucial to the normal development of the fetus. It surrounds the fetus, which acts as a mechanical buffer to balance the external pressure. It also contains a variety of nutrients and growth factors that are needed for the growth of the fetus [36]. The diversity in the composition of the AF provides a good source of research materials, providing opportunities for the assessment of fetal maturity, disease diagnosis, the discovery of biomarkers, etc. [37–39]. Therefore, scientific research on the composition of AF is greatly warranted by applying untargeted metabolomics. It is recognized that the composition of the AF changes with the growth of the fetus, but at present, little is known regarding the factors that affect the composition of the AF. In this study, we have applied an untargeted metabolomics method to identify as many minute molecular metabolites as possible in a selected cohort of AFs. Our findings revealed that the main metabolites in AF were amino acids, followed by organic acids, carbohydrates and fatty acids, which provided further insight into the composition of the AF. Nevertheless, the effects of several factors such as the gestational week, maternal age and fetal gender on the metabolic composition of AF remained inadequately understood.

Following the analysis of AF metabolic profile using an untargeted metabolomics approach, the findings were correlated with factors including fetal gender, maternal age, and gestational week. We found that there was no significant correlation between the AF metabolic profile and differences in the fetal gender, maternal age and gestational week within a small range of maternal age (21 to 40 years) and gestational week (17 to 22 weeks). However, several metabolite levels were affected. Our findings were consistent with the study by Graca et al. [40] that examining AF metabolism using Nuclear Magnetic Resonance (NMR) spectroscopy on 51 AF samples. Additionally, our findings revealed the metabolites that may be influenced by these three factors. Furthermore, the study by Orczyk-Pawilowicz et al. [21] has demonstrated the differences in the AF metabolic profile between the second and third trimester of pregnancy, suggesting that the gestational week may affect the constitutions of the AF if gestational week varies greatly. All in all, the influence of gestational week on the metabolic profile of AF is dependent on the range of gestational week.

There were several limitations to our study. Firstly, maternal BMI may influence the metabolic profile of AF but this was not explored. Secondly, sample sizes were small for several subgroup analyses. Nevertheless, our findings provide a basis for further research, which will shed light on how these factors may influence the AF metabolic profile and will generate opportunities for clinical application.

In summary, this study correlated the AF metabolic profile with several factors including detailed classifications of the gestational week, maternal age, and fetal gender. A small range of maternal age or gestational week did not significantly affect the AF metabolic profile but impacted several metabolite expressions. Gestational week, maternal age and fetal gender may affect the expression of some metabolites and thus, correlation of these factors in clinical studies is paramount. This study demonstrates a new approach in analyzing the metabolites in AF that guides the further study of biomarkers in pregnancy-related diseases.

## Data Availability

All authors had full access to the data and materials. Data is available from the authors upon reasonable request.
